# Exploring the relationship between co-worker and supervisor support, self- confidence, coping skills and burnout in residential aged care staff

**DOI:** 10.1186/s12912-022-00901-6

**Published:** 2022-06-01

**Authors:** Yin Siu Low, Sunil Bhar, Won Sun Chen

**Affiliations:** 1 Department of Psychological Sciences, School of Health Sciences, Swinburne University of Technology, Melbourne, Australia; 2 Department of Health Sciences and Biostatistics, School of Health Sciences, Swinburne University of Technology, Melbourne, Australia

**Keywords:** Professional care-givers, Occupational stress, Predictors

## Abstract

**Background:**

Staff working in residential aged care facilities face unique challenges and stressors in their workplaces which increase their risk for occupational burnout. Burnout in this workforce results in low job satisfaction, increased levels of absenteeism and poor retention rates. Given Australia’s ageing population and the demand for residential aged care staff, it is imperative to explore predictors of burnout in this cohort in order to help staff prevent and manage burnout.

**Methods:**

This study examined the extent to which co-worker and supervisor support, self-confidence and adaptive coping skills predicted burnout among residential aged care staff, after controlling for staff demographics, organisational climate and work patterns. One hundred and thirty three residential aged care staff across Australia were surveyed using online questionnaires measuring levels of co-worker and supervisor support, self- confidence, adaptive coping skills and burnout.

**Results:**

Regression analyses indicated that, overall, co-worker and supervisor support, self-confidence, and adaptive coping predicted each dimension of burnout (emotional exhaustion, depersonalization and personal accomplishment). After controlling for covariates and other predictors: confidence significantly predicted all three dimensions; support significantly predicted emotional exhaustion and depersonalization, and adaptive coping significantly predicted emotional exhaustion.

**Conclusion:**

These findings suggest that co-worker and supervisor support, self- confidence and adaptive coping skills need to be addressed to prevent and manage occupational burnout for residential aged care staff in Australia.

## Introduction

Australia’s population is ageing. The number of Australians aged 65 or older is estimated to increase from 15% in 2017 to between 21 and 23% in 2066 [1]. Older adults may require assistance in various aspects such as their daily living and social engagements, their personal care (dressing, eating and toileting), health care (medical, nursing, physiotherapy, dietetics and dentistry) and accommodation [2]. An estimate of 3.5 million people will require aged care services by 2050 and it is predicted that almost one million aged care workers need to be employed to sustain this growth [2].

Residential aged care (RAC) is for older adults who are not able to live at home and whose demands require greater care and support. Services included ongoing support and care with everyday tasks, personal care and 24-h care [3]. In 2020, there are 238,778 older adults in Australia who live in residential aged care facilities (RACFs) permanently and 65,709 older adults who receive respite care [3]. Older adults living in RACFs have complex physical comorbidities, chronic medical illnesses such as dementia [4], as well as mental health issues such as depression, phobias, anxiety and psychosis [5] and are highly dependent on care from staff members [6]. A total number of 153,854 direct care workers are employed in RACFs in Australia [7].

While supporting the care needs of these older adults, RAC staff experience a significant number of workplace stressors such as high workload and demands, insufficient staffing, lack of resources, violence from residents and conflicts with residents’ families [8, 9]. As such, RAC staff are at risk for burnout, which has been found to adversely impact on their well-being, resulting in high turnover rates and a reduction in the quality of care rendered to these residents [10, 11].

Hence, it is imperative to examine predictors of burnout in RAC staff to mitigate burnout, improve their well-being and to retain the aged care workforce. By examining and identifying predictors of burnout, interventions can be developed to precisely target these predictors to reduce burnout in such a workforce. Interventions that are precise in their targets of action are projected to not only reduce burnout, but also to result in improved client care.

### Occupational burnout syndrome

Occupational burnout has been conceptualised as a psychological syndrome due to chronic work-related interpersonal stressors [12]. Maslach and Jackson [13] posited that there are three key dimensions of such burnout: emotional exhaustion, depersonalization and reduced personal accomplishment. Emotional exhaustion refers to the depletion of one’s emotional capacity, depersonalization represents one’s cynicism and detachment from workplace issues, and reduced personal accomplishment refers to having a negative view of one’s work achievements [12, 13]. Maslach [14] interviewed staff working in the human services sector and found that that workers who reported high levels of occupational stress were emotionally drained, had developed negative feelings and attitudes towards their clients whom they were caring for and also struggled with their professional ability to execute a job meaningfully.

Individuals who experience burnout have been found to be less likely to remain in their work role and more likely to leave their work role, be absent from work, have physical sickness, suffer mental health symptoms; in setting with high rates of staff who experience burnout, there are high rates of turnover and greater intentions to quit among staff [15]. The prevalence of burnout in staff working in residential aged care facilities have been found to be higher than staff working in community settings [11, 16] suggesting that staff working in such facilities are prone to high levels of burnout.

Staff working in RACFs face unique challenges and stressors in their workplaces which increase their risks of occupational burnout. Organisational factors that predict burnout include low levels of staffing and work overload [8, 9], insufficient training and resources for the job role and poor remuneration [9]. Evers et al. [8] also found that the weekly working hours of RAC staff were positively associated with the emotional exhaustion dimension of burnout. Another study by Al Sabei et al. [17] examined job satisfaction as a moderator between work environment and turnover intentions for nursing staff employed in hospital settings. For staff who reported high levels of job satisfaction, there is less turnover intention when they are working in a conducive work environment [17]. In addition, demographics variables such as younger age, male staff and speaking English as a second language predicted higher burnout levels in RAC staff [18, 19]. Past researchers have also found gender differences in burnout levels in which male staff members have higher levels of depersonalization as compared to their female counterparts [18, 20]. This is likely due to the differences in coping styles where women tend to express their emotions and men tend to remove themselves and hide their emotions when under stress [21].

### Burnout and support, confidence and adaptive coping

Other predictors of burnout in RAC staff have been: support [22], confidence [23] and coping skills [24]. In a recent systematic review, Costello et al. [19] extracted data from seventeen studies and found that staff who felt unsupported experienced higher levels of burnout. Woodhead et al. [22] showed that support from supervisors, friends or family members were associated with lower levels of emotional exhaustion and greater personal accomplishment. Furthermore, Jeon et al. [25] found that support from management was significantly associated with lower levels of emotional exhaustion and depersonalization in RAC staff.

There is also growing evidence for a significant relationship between staff burnout levels and their confidence in managing older adults [23, 26]. In a recent mixed methods scoping review, Shrestha et al. [27] investigated caring self-efficacy in direct care workers employed in residential aged care settings and findings from their study showed that self-efficacy was negatively associated with burnout and work stress. In addition, Duffy et al. [23] examined the association of burnout and aged care staff's confidence in caring for older adults with dementia. The researchers found that levels of self-efficacy among staff were negatively associated with emotional exhaustion and depersonalization, and positively associated with personal accomplishment. Mackenzie and Peragine [26] investigated the impact of a self-efficacy training program for staff caring for residents with dementia. The self-efficacy training program focussed on aspects such as managing workplace conflicts among colleagues, addressing residents’ challenging behaviours and improving interactions with residents’ families [26]. Results from their study highlighted a reduction in burnout levels with staff experiencing greater personal accomplishments [26] which was consistent with past research by Brouwers et al. [28] who found that self-efficacy of school staff was positively correlated to personal accomplishment.

Coping skills are utilised by individuals in stressful circumstances where the demands surpass one’s personal resources. Adaptive coping refers to mental reasoning and behavioural strategies used to combat stressful events or overwhelming emotional states [29]. Adaptive coping acts as a protective factor against negative impacts of stressful life events and reduces the frequency of stressful events [29]. These behavioural and cognitive strategies assist individuals to reduce or tolerate internal and external demands [30]. Lee et al. [24] conducted a meta-analysis of seven studies; they found that coping strategies, especially problem-focused coping, decreased nurses’ emotional exhaustion and depersonalization [24]. Mackenzie et al. [31] found that a brief mindfulness-based stress reduction intervention for nurses and nurse aides reduced emotional exhaustion, hence providing further support for the importance of coping skills in reducing burnout.

### Aims and hypothesis of the study

International research has examined burnout in RAC settings in various countries [8, 9, 18, 20], however, there remains a paucity of research examining the extent to which support, confidence and adaptive coping predict burnout amongst Australian RAC staff. Further, given that some predictors—such as support, confidence and adaptive coping—are more easily modifiable at an individual level, it would also be important to examine their prediction of burnout, once controlling for more permanent or systemic factors such as demographics, organisational and work pattern characteristics. The first aim of this study was to examine the extent to which support, confidence and adaptive coping skills predicted dimensions of burnout, while controlling for organisational, demographic and work factors.

The second aim of this study was to identify the strongest unique predictors of burnout. The third aim of this study was to assess the levels of burnout, support, confidence and adaptive coping among the RAC staff working in Australia.

## Methods

### Sample

A sample of 133 RAC staff employed in RACFs across Australia were invited to participate in this study between September and December 2020. Researchers conducted a search on the Internet for RACFs and retrieved their email contact from publicly available online databases (https://www.gen-agedcaredata.gov.au/resources/access-data/2020/october/aged-care-service-list-30-june-2020). Facilities across Australia were contacted via email with a study poster attached, to be placed on RACFs staff noticeboards. The study poster comprised the survey link (www.embracesurvey.com) and QR code to the online questionnaire.

Inclusion criteria for staff participants were a) 18 years old or older, b) had sufficient English, c) were a paid employee of the facility, and d) provided direct care to residents.

Following a self-screen checklist, interested and eligible staff participated in the study by completing self-report questionnaires via the survey link. Participants in this study were reimbursed with a draw of $150 electronic gift card for their participation time.

Assuming a medium effect size, a significance level of 5%, and power of 80%, a sample size of 97 was sufficient Gpower (version 3.1.9.4) [32], after assuming a 20% drop out rate (sample for analysis = minimum of 77).

### Ethics approval

This study was approved by the Swinburne University of Technology Human Research Ethics Committee (SUHREC) (Reference no. 20203027–5106, 8 September 2020).

## Measures

### Materials

#### Burnout

Measures of burnout, support, confidence in managing aged care residents, adaptive coping skills, demographics and work environment were used in this study. Burnout. The Maslach Burnout Inventory Human Services Survey (MBI-HSS) [33] was used to assess burnout in workers employed in the human services settings. There were three dimensions of burnout measured in this scale: emotional exhaustion, depersonalization and personal accomplishment. Emotional exhaustion (EE) referred to a depletion of “emotional tanks” at work and the feeling that one has “nothing left to give” psychologically [12, 13]. Depersonalization (DP) referred to workers' cynical attitudes, negative feelings towards their clients’ issues and detachment from one’s workplace [12, 13]. Personal accomplishment (PA) described feelings of satisfaction with work accomplishments [12, 13]. The scale comprised 22 items. Using a 7- point likert scale (0 = never, 6 = often). Respondents indicated the extent to which each item described them with regards to their jobs. Sample items included “I feel emotionally drained from my work.”, “I have accomplished many worthwhile things in this job.” and “I don't really care what happens to some recipients. “High levels of emotional exhaustion and depersonalization coupled with low levels of personal accomplishment indicated burnout in a worker. The reported reliability for the survey was acceptable, with Cronbach’s Alpha coefficients of 0.71 for personal accomplishment, 0.79 for depersonalization and 0.90 for emotional exhaustion [23]. In this study, internal consistency Cronbach’s alpha for each of the burnout dimensions were as follows: emotional exhaustion Cronbach’s alpha = 0.94, depersonalization Cronbach’s alpha = 0.70, personal accomplishment Cronbach’s alpha = 0.65.

#### Support

Three subscales (cohesion, trust and support) from the Organizational Climate Questionnaire (OCQ) [34], were used to measure staff perception of support from co-workers and supervisors in the organization. Each subscale comprised of 5 items. For each item, respondents chose one out of five choices (where 1 = strongly disagree, 5 = strongly agree) to indicate whether they agreed or disagreed with each statement. Sample items included “People at work pitch in to help each other out.”, “I can count on my boss to keep the things I tell him confidential.” and “I can count on my boss to help me when I need it.” Scores across subscales were added to create a composite score for support, such that higher scores reflected higher levels of support. The reported reliability of the OCQ scale was high, with reported alpha coefficients ranging from 0.80 to 0.89 [34]. In this study, internal consistency Cronbach’s alpha of the composite of these subscales was high (α = 0.95).

#### Confidence

The Inventory of Geriatric Nursing Self-Efficacy (Self-efficacy measure) [26] was used to measure levels of confidence in managing geriatric nursing care challenges faced by professional caregivers. The inventory comprised 9 items including three items reflecting teamwork scenarios, three items reflecting resident scenarios and the other three items are family scenarios. Respondent indicated on a 7-point scale how confident they were in remaining calm, resolving the issue and achieving a positive result in relation to the scenarios (where 1 = not at all confident, 7 = very confident). Sample items included “You are extremely busy, you are behind in your work, and one of the residents is following you around and trying to grab your arm.” and “A colleague of yours is avoiding you for some reason. This is making your job difficult because you work closely with him.” Higher total scores indicated high levels of self-efficacy experienced by the individual. This measure was found to have good internal reliability with a Cronbach’s alpha of 0.96 and average item–total correlation of 0.83 [26]. In this study, the internal consistency of the self-efficacy scale, as measured by Cronbach’s alpha, was 0.93.

#### Adaptive coping

The adaptive coping subscale of the brief COPE questionnaire (brief COPE) [35]—a shortened version of the COPE inventory (COPE) [36] was used to measure adaptive coping. The subscale comprised a total of 16 items, with two items measuring each of the following aspects: active coping, planning, use of emotional support, use of instrumental support, positive reframing, acceptance, religion, and humour. Respondents use a four-point scale to indicate how frequently they employed these coping strategies (where 1 = I don’t usually do this at all, 4 = I usually do this a lot). Sample items include: “I've been concentrating my efforts on doing something about the situation I'm in.” and “I have been thinking hard about what steps to take.”, and “I've been getting emotional support from others”. The Cronbach’s alphas for the brief COPE subscales have been found to range from 0.50 to 0.90 [37]. In this study, the internal consistency of the adaptive coping subscale Cronbach’s alpha was 0.90. Demographics, work patterns and organisational climate were measured as covariates. Demographics characteristics included: gender (recorded 1 = female, 2 = male), education (recorded 1 = postgraduate, 2 = undergraduate, 3 = Technical and Further Education (TAFE) diploma or certificate, 4 = Year 12, 5 = Year 11, 6 = Year 10, 7 = Year 9, 8 = Year 8, 9 = No school) and languages (recorded 1 = English, 2 = Italian, 3 = Greek, 4 = Cantonese, 5 = Arabic, 6 = Mandarin, 7 = Vietnamese, 8 = Other). Work patterns characteristics included in this study were: schedule (recorded 1 = regular day-time shift, 2 = rotating shift pattern, 3 = other) and workplace training received in the past 12 months (1 = No, 2 = Yes).

Three subscales of the Organizational Climate Questionnaire (OCQ) [34]—autonomy, pressure and recognition—were used to  measure staff perception of work independence, workload and skill recognition in the organization. Each subscale comprised of 5 items. For each item, respondents were required to choose one out of five choices (where 1 = strongly disagree, 5 = strongly agree) to indicate whether they agree or disagree with each statement. Items included “I determine my own work procedure.”, “I have too much work and too little time to do it in.” and “My boss is quick to recognize good performance.” The 15-items were summed to obtain organizational climate functioning scores, which ranged from 5 to 75 where higher scores indicated higher levels of positive organizational climate functioning. In this study, internal consistency Cronbach’s alpha was.60.

## Statistical analyses

Data were summarised using descriptive statistics, such as mean, standard deviation, median and range, for continuous variables while frequency and percentage were presented for categorical variables. Internal consistencies were described using Cronbach’s alpha.

Hierarchical regression was conducted to examine the extent to which the purported predictor variables predicted burnout dimensions after controlling for covariates. Three regression analyses were conducted each with a dimension of burnout as the dependent variable. Covariates were demographics, work patterns and organisational climate (Step 1); predictor variables were support, confidence and adaptive coping (Step 2).

Assumptions relating to multicollinearity, outliers, normality, linearity, homoscedasticity, independence of residuals were tested. All assumptions were met. A *p*- value of < 0.05 was deemed statistically significantly for all two-sided tests. The analyses was conducted using IBM SPSS Statistics version 27 [38].

## Results

### Sample

The study was conducted across Australian RACFs. The sample comprised 133 RAC staff. Of these, 1 RAC staff was excluded from this study due to missing data, leaving a final sample of 133 participants (mean age is 44.1 years, SD = 13.9). Their mean length of time employed in the aged care industry was 115.4 months (SD = 112.40). Their mean length of time employed in their current job was 55.40 months (SD = 59.40). The mean working hours in a week for the sample was 34.90  (SD = 12.10).

The sociodemographic variables and educational qualifications of the participants are presented in Tables 1 and 2. Participants were mostly female, from Oceania (Australia and New Zealand), employed in a full-time permanent capacity with a regular day-time shift work schedule. Most participants worked as personal care attendants, followed by management and administration roles. Most participants had a vocational diploma or certificate, while approximately a quarter had either a postgraduate university degree or an undergraduate university degree. Nearly all participants had undergone training in the workplace in the last 12 months.

**Table 1:** Descriptive statistics of participants biographical information (*N* = 133)

	Mean	Standard deviation	Range (Min–max)
Age	44.10	13.90	19–81
Length of time employed in aged care (months)	115.40	112.40	1–500
Length of time in current job (months)	55.40	59.40	1–303
Hours worked in week	34.90	12.10	5–76

**Table 2:** Sociodemographic, work characteristics and educational qualifications of participants

Variable	Number of participants	Percentage
Gender		
Male	12	9.0
Female	121	91.0
Job title		
Management	30	22.6
Administration	14	10.5
Spiritual care work	1	0.8
Care co-ordinator	9	6.8
Nurse practitioner	0	0.0
Registered nurse	12	9.0
Enrolled nurse	9	6.8
Personal care attendant	41	30.8
Allied health professionals	5	3.8
Allied health assistants	2	1.5
Other	10	7.5
Work classification		
Full-time permanent	57	42.9
Permanent part-time	63	47.4
Casual/contract full-time	4	3.0
Casual/contract part-time	9	6.8
Work schedule		
Regular day-time shift	88	66.2
Rotating shift pattern	27	20.3
Other	18	13.5
Work training (last 12 months)		
No training provided	11	8.3
Training provided	122	91.7
Continents		
Asia	20	15.0
Africa	4	3.0
Oceania	97	72.9
North America	1	0.8
Europe	11	8.3
Education		
Postgraduate degree	37	27.8
Undergraduate degree	29	21.8
TAFE Diploma or certificate	59	44.4
High school	8	6.0
Languages spoken		
English	108	81.2
Mandarin	4	3.0
Vietnamese	1	0.8
English language competency		
Excellent	123	92.5
Good	10	7.5

Table 3 presents the means, standard distribution and ranges of predictor and dependent variable. Results showed low to moderate level of support, confidence, adaptive coping, as well as low to moderate level of emotional exhaustion, depersonalization and personal accomplishment.

**Table 3:** Levels of burnout, support, confidence and adaptive coping in a sample of aged care staff (*N* = 133)

Variable	Minimum	Maximum	Mean	SD
Emotional exhaustion	1	54	24. 99	14.89
Depersonalisation	0	27	5.48	5.57
Personal accomplishment	19	48	38.64	6.27
Support	17	75	53.50	13.05
Confidence	9	63	51.08	9.37
Adaptive coping	16	63	37.16	10.45

Bivariate association between predictors and burnout Table 4 shows the Pearson’s correlation coefficients between predictor variables and the three dimensions of burnout. The findings showed that support was significantly negatively correlated with emotional exhaustion and depersonalisation, while significantly positively correlated to personal accomplishment. Higher levels of support were correlated with lower levels of emotional exhaustion depersonalisation and higher levels of personal accomplishment. Confidence was significantly negatively correlated to depersonalisation and positively correlated with personal accomplishment. This suggests that lower levels of confidence were correlated to higher levels of depersonalisation while higher levels of confidence were correlated to higher levels of personal accomplishment. There was no significant correlation found between adaptive coping and all three dimensions of burnout.

**Table 4:** Pearson’s correlation coefficients between support, confidence, adaptive coping and burnout (emotional exhaustion, depersonalisation and personal accomplishment (*N* = 133)

Variable	Emotional exhaustion	Depersonalisation	Personal accomplishment	Support	Confidence
Emotional exhaustion					
Depersonalisation	.68**				
Personal accomplishment	-.31**	-.39**			
Support	-.50**	-.34**	.18*		
Confidence	-.17	-.22*	.26**	.02	
Adaptive coping	.15	.13	.08	-.02	-.03

^*^p < .05; **p < .01; ***p < .001

### Hierarchical *r*egression

#### Emotional exhaustion

Table 5 presents the results of the hierarchical regression analyses. Age, gender, education, language, organisational climate, hours, schedule, training were entered at Step 1, explaining 10% of the variance in emotional exhaustion. After entry of support, confidence, adaptive coping at Step 2, the total variance explained by the model as a whole was 38.4%, F (11, 121) = 6.87, *p* < 0.001. The support, confidence, adaptive coping measures explained an additional 28% of the variance in emotional exhaustion after controlling for age, gender, education, language, organisational climate, hours, schedule, training, R squared change = 0.28, F change (3, 121) = 18.51, *p* < 0.001. More specifically, higher levels of support significantly predicted lower emotional exhaustion scores (*p* < 0.001) and lower depersonalization scores (*p* < 0.001); this prediction was not significant for personal accomplishment scores once demographics, organisational climate and work patterns were controlled.

**Table 5:** Hierarchical regression models showing factors associated with the Maslach Burnout Inventory (Human Services) Scale-22 items (MBI-HSS) Emotional Exhaustion, Depersonalisation and Personal Accomplishment subscale scores

Step	Independent variables		MBI_EE				MBI_DP				MBI_PA		
		*R*^*2*^	Change in *R*^*2*^		*P*-value	*R*^*2*^	Change in *R*^*2*^		*P*-value	*R*^*2*^	Change in *R*^*2*^		*P*-value
1	Age, gender, education, language, organisational climate, hours, schedule, training	.100	.102		> 0.05	.108	.108		> 0.05	.095	.095		> 0.05
2	Added support, confidence, adaptive coping	.384	.283		< 0.001	.246	.138		< 0.001	.168	.073		< 0.05
Final model		B (s.e)	β	*P*-value	95% CI	B (s.e.)	β	*P*-value	95% CI	B (s.e.)	β	*P*-value	95% CI
	Age	-0.03 (0.09)	-0.03	0.74	-0.20, 0.14	-0.10 (0.04)	-0.25	0.01	-0.17, -0.03	0.04 (0.04)	0.08	0.41	-0.05, 0.12
	Gender	-1.18 (3.77)	-0.02	0.76	-8.65, 6.29	1.25 (1.56)	0.06	0.43	-1.85, 4.34	-0.66 (1.85)	-0.03	0.72	-4.32, 2.99
	Education	-0.69 (1.06)	-0.05	0.52	-2.79, 1.41	-0.02 (0.44)	-0.00	0.97	-0.89, 0.85	0.35 (0.52)	0.06	0.50	-0.68, 1.38
	Language	-0.86 (0.47)	-0.15	0.07	-1.78, 0.07	-0.28 (0.19)	-0.13	0.15	-0.67, 0.10	0.24 (0.23)	0.10	0.31	-0.22, 0.69
	Organisational climate	0.38 (0.20)	0.18	0.06	-0.01, 0.76	0.10 (0.08)	0.13	0.22	-0.06, 0.26	0.13 (0.10)	0.15	0.18	-0.06, 0.32
	Hours	0.11 (0.10)	0.09	0.29	-0.09, 0.30	0.03 (0.04)	0.06	0.50	-0.05, 0.11	-0.02 (0.05)	-0.04	0.69	-0.11, 0.08
	Schedule	-1.98 (1.74)	-0.10	0.26	-5.42, 1.46	-0.45 (0.72)	-0.06	0.54	-1.87, 0.98	0.47 (0.85)	0.05	0.59	-1.22, 2.15
	Training	-6.12 (4.05)	-0.11	0.13	-14.14, 1.90	-1.53 (1.68)	-0.08	0.37	-4.85, 1.90	4.14 (1.98)	0.18	0.04	0.21, 8.06
	Support	-0.72 (0.11)	-0.63	< 0.001	-0.93, -0.50	-0.18 (0.05)	-0.41	< 0.001	-0.27, -0.09	0.06 (0.05)	0.12	0.27	-0.05, 0.16
	Confidence	-0.30 (0.12)	-0.19	0.02	-0.53, -0.06	-0.11 (0.05)	-0.18	0.03	-0.21, -0.01	0.18 (0.06)	0.27	0.00	0.06, 0.30
	Adaptive coping	0.27 (0.11)	0.19	0.02	0.05, 0.48	0.07 (0.05)	0.13	0.15	-0.02, 0.16	0.02 (0.05)	0.04	0.67	-0.08, 0.13

The regression models were planned to include (if significantly associated) demographics, organisational climate and work patterns (Step 1) and predictors of burnout: support, confidence and adaptive coping (Step 2). B, unstandardised coefficient; β, standardised coefficient; CI, confidence interval

#### Depersonalization

Age, gender, education, language, organisational climate, hours, schedule, training were entered at Step 1, explaining 10.8% of the variance in depersonalization. After entry of support, confidence, adaptive coping at Step 2, the total variance explained by the model as a whole was 25%, F (11, 121) = 3.59, *p* < 0.001. The support, confidence, adaptive coping measures explained an additional 14% of the variance in depersonalization after controlling for age, gender, education, language, organisational climate, hours, schedule, training, R squared change = 0.14, F change (3, 121) = 7.39, *p* < 0.001. In particular, higher levels of confidence significantly predicted lower emotional exhaustion scores (*p* < 0.05), lower depersonalization scores (*p* < 0.05) and higher personal accomplishment scores (*p* < 0.001) once all other variables were controlled.

#### Personal accomplishment

Age, gender, education, language, organisational climate, hours, schedule, training were entered at Step 1, explaining 10% of the variance in personal accomplishment. After entry of support, confidence, adaptive coping at Step 2, the total variance explained by the model as a whole was 17 %, F (11, 121) = 2.222, *p* < .05. The support, confidence, adaptive coping measures explained an additional 7% of the variance in personal accomplishment after controlling for age, gender, education, language, organisational climate, hours, schedule, training, R squared change= .07, F change (3, 121) = 3.56, *p* < .05. In addition, higher levels of adaptive coping significantly predicted lower emotional exhaustion scores (*p* < 0.05); the prediction was not significant for depersonalization and personal accomplishment scores once all other variables were controlled.

## Discussion

The aims of the study were to examine the extent to which support, confidence and adaptive coping in RAC staff employed in Australia predict their occupational burnout. This study found that support, confidence and adaptive coping were predictors of burnout in RAC staff, even after controlling for demographics, work patterns and organisational climate. This finding is discussed in detail below.

First, higher levels of support significantly predicted lower emotional exhaustion and lower depersonalization levels once demographics, organisational climate and work patterns were controlled. The role of support in occupational burnout has been well-documented in the literature [22, 25]. RAC staff who received greater levels of support from their managers experienced lower levels of emotional exhaustion and depersonalization [25].

The findings from this study not only supported the results of [25] and [22]; they also further emphasised the importance of the role of support even after more permanent aspects of the workplace such as demographics, organisational and work pattern characteristics were controlled.

Second, higher levels of confidence significantly predicted lower emotional and depersonalization levels and higher personal accomplishment levels while controlling for covariates. Similar to Shrestha et al. [27]’s mixed methods scoping review study, this study found that higher self-confidence in RAC staff were negatively associated with burnout levels. Results from our study supported findings from Duffy et al. [23]’s study where greater levels of self-efficacy in care staff had significantly contributed to lower levels of emotional exhaustion and depersonalization and higher levels of personal accomplishment [23]. Findings from this study were also consistent with past research by [26] and Brouwers et al. [28] where confidence levels of staff members were found to significantly predict personal accomplishment. Hence, the role of confidence in RAC staff was a vital component in predicting occupational burnout even after controlling for demographics, organisational and work patterns characteristics.

Third, higher levels of adaptive coping significantly predicted lower emotional exhaustion levels once demographics, organisational climate and work patterns were controlled and this finding was consistent with past research of the role of coping and burnout levels. The findings for coping strategies supported the results of Lee et al. [24] and Mackenzie et al. [31]. Unlike some studies [18, 20], this current study controlled for gender, therefore gender differences in burnout and coping styles were not observed.

Since the current study controlled for covariates such as demographics, organisational climate and work patterns, the results provided a strong evidence that adaptive coping significantly predicted lower levels of emotional exhaustion in RAC staff. Hence, individuals who utilise adaptive coping skills such as problem-solving and knowledge seeking are likely to cope better during stressful life events [29]. The type of coping strategies used by individuals is vital as it impacts on one’s psychological and physical health [29]. Therefore, it is important to utilise adaptive coping strategies as there is an indirect impact on RAC staff’s wellbeing which may also adversely impact on the quality of care provided to residents.

The clinical implication of this study is that in order to better manage occupational burnout in RAC staff working in RACFs, it is imperative for future intervention programs to incorporate support, confidence and adaptive coping skills in order to help them reduce or manage occupational burnout. By fostering greater workplace support from supervisors and colleagues, equipping RAC staff with adaptive coping skills can further contribute to staff members’ confidence levels, which will result in better management of occupational burnout in this cohort.

This study had several limitations. The focus on a single country in this study limits the ability to generalise these current findings to other countries. Self-selection bias may also be present in this study as staff members who completed the survey may be different from other staff members who have decided not to participate in this burnout study. Furthermore, causality has not been established in this study. It is also not known whether these predictors of burnout apply equally to staff members employed in different geriatric settings such as inpatient wards in hospitals. Comparative studies, particularly, among gender differences in burnout and coping styles can also be undertaken in the near future.

## Conclusion

Burnout can reduce RAC staff wellbeing and may impact adversely on the quality of care such staff provide to residents. The current study examined support, confidence and adaptive coping as predictors of burnout in RAC staff working in Australia. It found that support, confidence and adaptive coping significantly predicted burnout symptoms in RAC staff, after controlling for staff demographics, organisational climate and work patterns. Taken together, our findings highlight the role of support, confidence and adaptive coping skills and how these predictors need to be addressed in future interventions for occupational burnout among RAC staff in Australia.

## Data Availability

The datasets used and/or analysed during the current study are available from the corresponding author on reasonable request.

## Abbreviations

RAC: Residential aged care
RACFs: Residential aged care facilities

## References

[1] Australian Bureau of Statistics. (2018). Population projections, Australia, 2017 (base)-2066 (cat. no. 3222.0). https://www.abs.gov.au/

[2] Productivity Commission. (2011). Caring for older Australians: Final inquiry report (Report Number 53). https://www.pc.gov.au/

[3] Productivity Commission. (2021). Section 14 Aged Care Services - Report on Government Services 2021. https://www.gen-agedcaredata.gov.au/www_aihwgen/media/Productivity-Commission/rogs-2021-partf-section14-aged-care-services.pdf

[4] Dyer SM, Gnanamanickam ES, Liu E, Whitehead C, Crotty M (2018). Diagnosis of dementia in residential aged care settings in Australia: An opportunity for improvements in quality of care?. Australas J Ageing.

[5] Amare AT, Caughey GE, Whitehead C, Lang CE, Bray SC, Corlis M, Visvanathan R, Wesselingh S, Inacio MC (2020). The prevalence, trends and determinants of mental health disorders in older Australians living in permanent residential aged care: Implications for policy and quality of aged care services. Aust N Z J Psychiatry.

[6] Pitfield C, Shahriyarmolki K, Livingston G (2011). A systematic review of stress in staff caring for people with dementia living in 24-hour care settings. Int Psychogeriatr.

[7] Mavromaras, K., Knight, G., Isherwood, L., Crettenden, A., Flavel, J., Karmel, T., Moskos, M., Smith, L., Walton, H., & Wei, Z. (2017). 2016 National Aged Care Workforce Census and Survey – The Aged Care Workforce, 2016. https://agedcare.health.gov.au/sites/g/files/net1426/f/documents/03_2017/nacwcs_final_report_290317.pdf

[8] Evers W, Tomic W, Brouwers A (2002). Aggressive behaviour and burnout among staff of homes for the elderly. Int J Ment Health Nurs.

[9] Yeatts DE, Seckin G, Shen Y, Thompson M, Auden D, Cready CM (2018). Burnout among direct-care workers in nursing homes: Influences of organisational, workplace, interpersonal and personal characteristics. J Clin Nurs.

[10] Hunter PV, Hadjistavropoulos T, Thorpe L, Lix LM, Malloy DC (2016). The influence of individual and organizational factors on person-centred dementia care. Aging Ment Health.

[11] McHugh MD, Kutney-Lee A, Cimiotti JP, Sloane DM, Aiken LH. Nurses’ widespread job dissatisfaction, burnout, and frustration with health benefits signal problems for patient care. Health Aff. 2011;30(2):202–10. 10.1377/hlthaff.2010.0100. 10.1377/hlthaff.2010.0100 PMC3201822 21289340

[12] Maslach C, Schaufeli WB, Leiter MP (2001). Job burnout. Annu Rev Psychol.

[13] Maslach C, Jackson SE (1981). The measurement of experienced burnout. J Organ Behav.

[14] Maslach C. Burn-Out. Human Behavior. 1976;5(9):16–22.

[15] Karantzas GC, Mellor D, McCabe MP, Davison TE, Beaton P, Mrkic D (2012). Intentions to quit work among care staff working in the aged care sector. Gerontologist.

[16] Boekhorst S, Willemse B, Depla MF, Eefsting JA, Pot AM (2008). Working in group living homes for older people with dementia: The effects on job satisfaction and burnout and the role of job characteristics. Int Psychogeriatr.

[17] Al Sabei SD, Labrague LJ, Miner Ross A, Karkada S, Albashayreh A, Al Masroori F, Al Hashmi N (2020). Nursing work environment, turnover intention, job burnout, and quality of care: the moderating role of job satisfaction. J Nurs Scholarsh.

[18] Costello H, Cooper C, Marston L, Livingston G (2019). Burnout in UK care home staff and its effect on staff turnover: MARQUE English national care home longitudinal survey. Age Ageing.

[19] Costello H, Walsh S, Cooper C, Livingston G. A systematic review and meta-analysis of the prevalence and associations of stress and burnout among staff in long-term care facilities for people with dementia. International Psychogeriatrics. 2018;1-14. 10.1017/S1041610218001606 10.1017/S1041610218001606 30421691

[20] Canadas-De la Fuente GA, Vargas C, San Luis C, Garcia I, Canadas GR, De la Fuente EI (2015). Risk factors and prevalence of burnout syndrome in the nursing profession. Int J Nurs Stud.

[21] Purvanova RK, Muros JP (2010). Gender differences in burnout: a meta-analysis. J Vocat Behav.

[22] Woodhead EL, Northrop L, Edelstein B (2016). Stress, social support, and burnout among long-term care nursing staff. J Appl Gerontol.

[23] Duffy B, Oyebode JR, Allen J (2009). Burnout among care staff for older adults with dementia. Dementia.

[24] Lee HF, Kuo CC, Chien TW, Wang YR (2016). A meta-analysis of the effects of coping strategies on reducing nurse burnout. Appl Nurs Res.

[25] Jeon YH, Luscombe G, Chenoweth L, Stein-Parbury J, Brodaty H, King M, Haas M (2012). Staff outcomes from the caring for aged dementia care resident study (CADRES): a cluster randomised trial. Int J Nurs Stud.

[26] Mackenzie CS, Peragine G (2003). Measuring and enhancing self-efficacy among professional caregivers of individuals with dementia. Am J Alzheimers Dis Other Demen.

[27] Shrestha S, Alharbi RJ, Wells Y, While C, Rahman MA (2021). Caring self-efficacy of direct care workers in residential aged care settings: a mixed methods scoping review. Geriatr Nurs.

[28] Brouwers A, Evers WJG, Tomic W (2001). Self-efficacy in eliciting social support and burnout among secondary-school teachers. J Appl Soc Psychol.

[29] Holahan CJ, Ragan JD, Moos RH. Stress. Reference Module in Neuroscience and Biobehavioral Psychology. 2017;485-493. 10.1016/B978-0-12-809324-5.05724-2.

[30] Lazarus R, Folkman S (1984). Stress, Appraisal, and Coping.

[31] Mackenzie CS, Poulin PA, Seidman-Carlson R (2006). A brief mindfulness-based stress reduction intervention for nurses and nurse aides. Appl Nurs Res.

[32] Faul F, Erdfelder E, Lang A-G, Buchner A (2007). G*Power 3: a flexible statistical power analysis program for the social, behavioural, and biomedical sciences. Behav Res Methods.

[33] Maslach C, Jackson S. Maslach Burnout Inventory-Human Services Survey. Mind Gareden, Inc. Retrieved from http://www.mindgarden.com.

[34] Koys DJ, DeCotiis TA (1991). Inductive measure of psychological climate. Human Relations.

[35] Carver CS. You want to measure coping but your protocol’s too long: consider the Brief COPE. Int J Behav Med. 1997;4(1):92–100. 10.1207/s15327558ijbm0401_6. 10.1207/s15327558ijbm0401_6 16250744

[36] Carver CS, Scheier MF, Weintraub JK (1989). Assessing coping strategies: a theoretically based approach. J Pers Soc Psychol.

[37] Mohanraj R, Jeyaseelan V, Kumar S, Mani T, Rao D, Murray KR, Manhart LE (2015). Cultural adaptation of the Brief COPE for persons living with HIV/AIDS in southern India. AIDS Behav.

[38] IBM Corp (2020). IBM SPSS Statistics for Windows, Version 2.70.

